# Timing Calibration and Windowing Technique Comparison for Lightning Mapping Arrays

**DOI:** 10.1029/2020EA001523

**Published:** 2021-07-08

**Authors:** Brian M. Hare, Harald Edens, Paul Krehbiel, William Rison, O. Scholten, S. Buitink, A. Corstanje, H. Falcke, J. R. Hörandel, Tim Huege, G. K. Krampah, P. Mitra, K. Mulrey, Anna Nelles, Hershal Pandya, J. P. Rachen, S. Thoudam, T. N. Trinh, S. ter Veen, Tobias Winchen

**Affiliations:** ^1^ Kapteyn Astronomical Institute University of Groningen Groningen The Netherlands; ^2^ New Mexico Tech Socorro NM USA; ^3^ Interuniversity Institute for High‐Energy Vrije Universiteit Brussel Brussels Belgium; ^4^ Department of Astrophysics/IMAPP Radboud University Nijmegen Nijmegen The Netherlands; ^5^ Astrophysical Institute Vrije Universiteit Brussel Brussels Belgium; ^6^ NIKHEF Amsterdam The Netherlands; ^7^ Netherlands Institute of Radio Astronomy (ASTRON) Dwingeloo The Netherlands; ^8^ Institute for Astroparticle Physics (IAP) Karlsruhe Institute of Technology (KIT) Karlsruhe Germany; ^9^ DESY Zeuthen Germany; ^10^ ECAP Friedrich‐Alexander‐University Erlangen‐Nürnberg Erlangen Germany; ^11^ Department of Physics Khalifa University Abu Dhabi UAE; ^12^ Department of Physics School of Education Can Tho University Campus II Can Tho Vietnam; ^13^ Max‐Planck‐Institut für Radioastronomie Bonn Germany

## Abstract

Since their introduction 22 years ago, lightning mapping arrays (LMA) have played a central role in the investigation of lightning physics. Even in recent years with the proliferation of digital interferometers and the introduction of the LOw Frequency ARray (LOFAR) radio telescope, LMAs still play an important role in lightning science. LMA networks use a simple windowing technique that records the highest pulse in either 80 μs or 10 μs fixed windows in order to apply a time‐of‐arrival location technique. In this work, we develop an LMA‐emulator that uses lightning data recorded by LOFAR to simulate an LMA, and we use it to test three new styles of pulse windowing. We show that they produce very similar results as the more traditional LMA windowing, implying that LMA lightning mapping results are relatively independent of windowing technique. In addition, each LMA station has its GPS‐conditioned clock. While the timing accuracy of GPS receivers has improved significantly over the years, they still significantly limit the timing measurements of the LMA. Recently, new time‐of‐arrival techniques have been introduced that can be used to self‐calibrate systematic offsets between different receiving stations. Applying this calibration technique to a set of data with 32 ns uncertainty, observed by the Colorado LMA, improves the timing uncertainty to 19 ns. This technique is not limited to LMAs and could be used to help calibrate future multi‐station lightning interferometers.

## Key Points

- The LOFAR telescope can be used to emulate and explore the operation of LMA networks
- Different, new, windowing techniques for LMAs are developed and compared
- A timing calibration technique for LMAs is developed and presented

## 1 Introduction

Since their introduction 22 years ago, lightning mapping arrays (LMAs) have played a central role in investigating lightning physics and storm electrification processes (Rison et al., 1999). Even in recent years with the proliferation of higher‐time resolution digital interferometers (Stock et al., 2014) and the introduction of the LOw Frequency ARray (LOFAR) radio telescope (Hare et al., 2018, 2019; van Haarlem et al., 2013), LMAs still play an important role in lightning science due to being relatively easy to deploy, covering an area larger than an interferometer, and being able to detect lightning with significantly greater efficiency and detail than long‐range lightning detection networks.

LMA networks use a simple windowing technique that records the highest pulse in fixed time windows, either 80 μs or 10 μs in length, in order to apply a time‐of‐arrival location technique. Such a windowing scheme could potentially be improved, as high‐amplitude pulses that should be locatable often occur in the same time window, either at all or some of the stations, and/or with different peak amplitudes and being selected, in which case one or more pulses are not detected. This happens less often for 10 μs windows, but TOA data for the narrower windowing requires substantially longer times to process and is still affected to some extent by pulse overlap. Different windowing techniques may produce different lightning images, potentially leading to different physics interpretations.

In this study, we explore several different windowing techniques, and how they affect the imaged source locations. This study is conducted with an LMA‐emulator that uses lightning data recorded by LOFAR to simulate an LMA. We compare the results of three new styles of windowing to traditional 80 μs LMA windowing for two lightning flashes. One of which is close to LOFAR, one of which is more distant. We also apply new time‐of‐arrival techniques for self‐calibrating systematic offsets in LOFAR observations to develop an algorithm that corrects small remnant systematic timing differences between LMA stations. This algorithm can improve the timing accuracy of a set of data collected by the Colorado LMA (COLMA) (which typically has 25 ns uncertainty) from 32 to 19 ns. This technique is very instrument‐agnostic, and could be applied to future multi‐station interferometers.

## 2 Lightning Mapping Arrays

An LMA generally consists of 8–16 or more stations, and accurately measures the arrival times of impulsive VHF radiation events. The signals are received in a 6 MHz bandwidth in a locally unused television channel, with the arrival times and window boundaries for each second derived from the 1 pps (pulse per second) signals of a GPS receiver. The logarithmically detected signals are digitized at a 25 MHz rate with 16‐bit precision and processed in an on‐board field programmable gate array to determine the peak event in successive 80– or 10 μs time windows. Peak values above a floating noise threshold are saved to an output file. A subset of the data stream is decimated to 400 μs intervals and communicated via cellular data links to a central computer for real‐time processing and display. The full 80 μs data is post‐processed either daily or as needed depending on available cell data speeds. The times of the processing windows are fixed to align with the start and end of a second, and all processing is done on a second‐by‐second basis. The noise threshold floats such that there are about 1,000 pulses recorded in a 1 s period (Thomas et al., 2004).

The LMA detections have two main sources of timing uncertainty. First, each peak value has a random uncertainty of 12 ns rms, due to the peak time values being quantized to 40 ns time values by the digitizer. This quantization effect represents the minimum possible timing uncertainty of a network. The other uncertainty concerns the one second time interval. In particular, there is a ≃10–20 ns uncertainty in the timing of the GPS 1 pps pulses from the GPS receiver, and a random 0–40 ns delay until the time of the next 25 MHz clock pulse that defines the start of the one second interval. The timing uncertainty changes from second to second, but is systematic for a given second and is different for each station.

Each LMA source has a reduced chi‐square goodness of fit given by

(1) $$\chi_{\nu }^{2}=\frac{1}{N_{a}-4}\sum_{j}\frac{{\left(M_{j}-t_{j}\right)}^{2}}{\sigma_{\epsilon }^{2}}$$

where the sum is over each participating station. *N*_*a*_ is number of stations, and (*N*_*a*_ − 4) is the number of degrees of freedom *ν* in the solution. *M*_*j*_ is the modeled arrival time at the *j*th antenna, determined from the distance of the source from the station in question. *t*_*j*_ is the measured arrival time at station *j*, and *σ*_*ϵ*_ is the rms timing uncertainty of the network, which can be estimated from the chi‐square distributions of processed data. For current networks the timing uncertainty is about 25 ns rms. For a given source, its chi‐square fit can also be expressed as a RMS timing uncertainty, given by

(2) $$RMS=\sigma_{\epsilon }\sqrt{\chi_{\nu }^{2}{|}_{i}},$$

where $\chi_{\nu }^{2}{|}_{i}$ is the reduced chi‐square value of the particular source in question.

Following the normal procedure for LMA networks, we use the distribution of reduced chi‐square values to estimate the rms timing uncertainty *σ*_*ϵ*_ of different LMA data sets. This is done by plotting the distribution of the $\chi_{\nu }^{2}$ for different degrees of freedom, and adjusting *σ*_*ϵ*_ until an agreement with the theoretical chi‐square distribution is obtained. The resulting *σ*_*ϵ*_ is then the timing uncertainty of the data set. Using this procedure, the timing uncertainty of the Colorado Lightning Mapping Array (COLMA) data used in this work is about 32 ns, where COLMA typically has a timing uncertainty around 25 ns.

## 3 LOFAR and the LMA‐Emulator

In order to investigate different windowing techniques, we use continuously recorded VHF observations of two lightning flashes collected by LOFAR to emulate an LMA. We refer to this as an LMA‐emulator. The benefit of such an emulator is that LOFAR saves five seconds of time series data for each trigger. Thus, the pipeline that each LMA station applies to its data in an online fashion can be applied to the LOFAR data as an off‐line process, allowing us easily explore different aspects of the LMA online processing, such as the windowing technique. In addition, LOFAR has random timing uncertainties better than 1 ns, and we have developed an algorithm to calibrate out systematic timing differences between LOFAR stations. The longest LOFAR baselines are comparable to that of an LMA, up to 100 km (Hare et al., 2018, 2019; van Haarlem et al., 2013).

**Figure 1:**
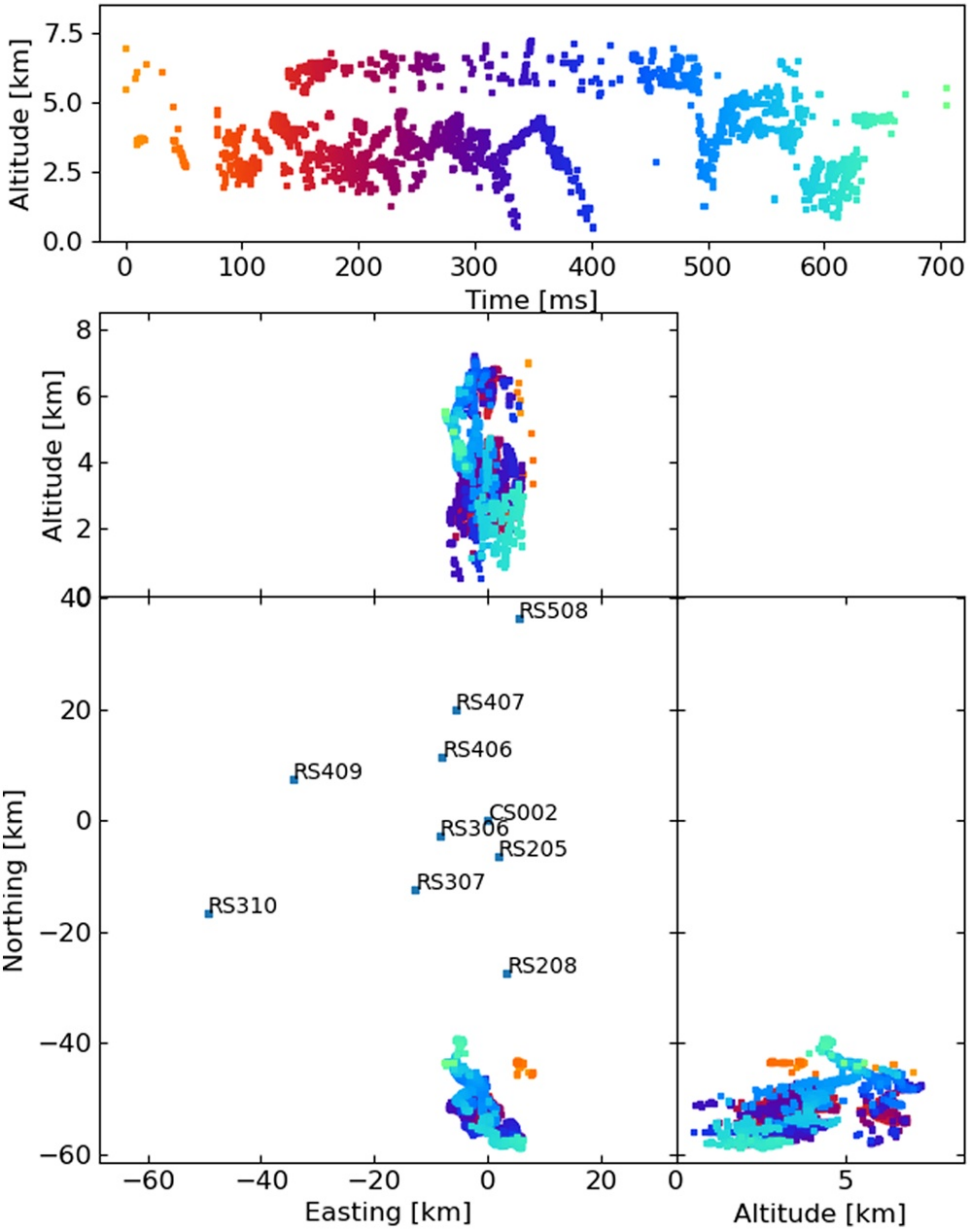
The 2018 lightning flash was mapped by the LOFAR LMA emulator using the traditional LMA windowing technique, along with the used LOFAR stations. Showing sources that have eight or more participating stations and a chi‐square value better than two (RMS < 21 ns).

**Figure 2:**
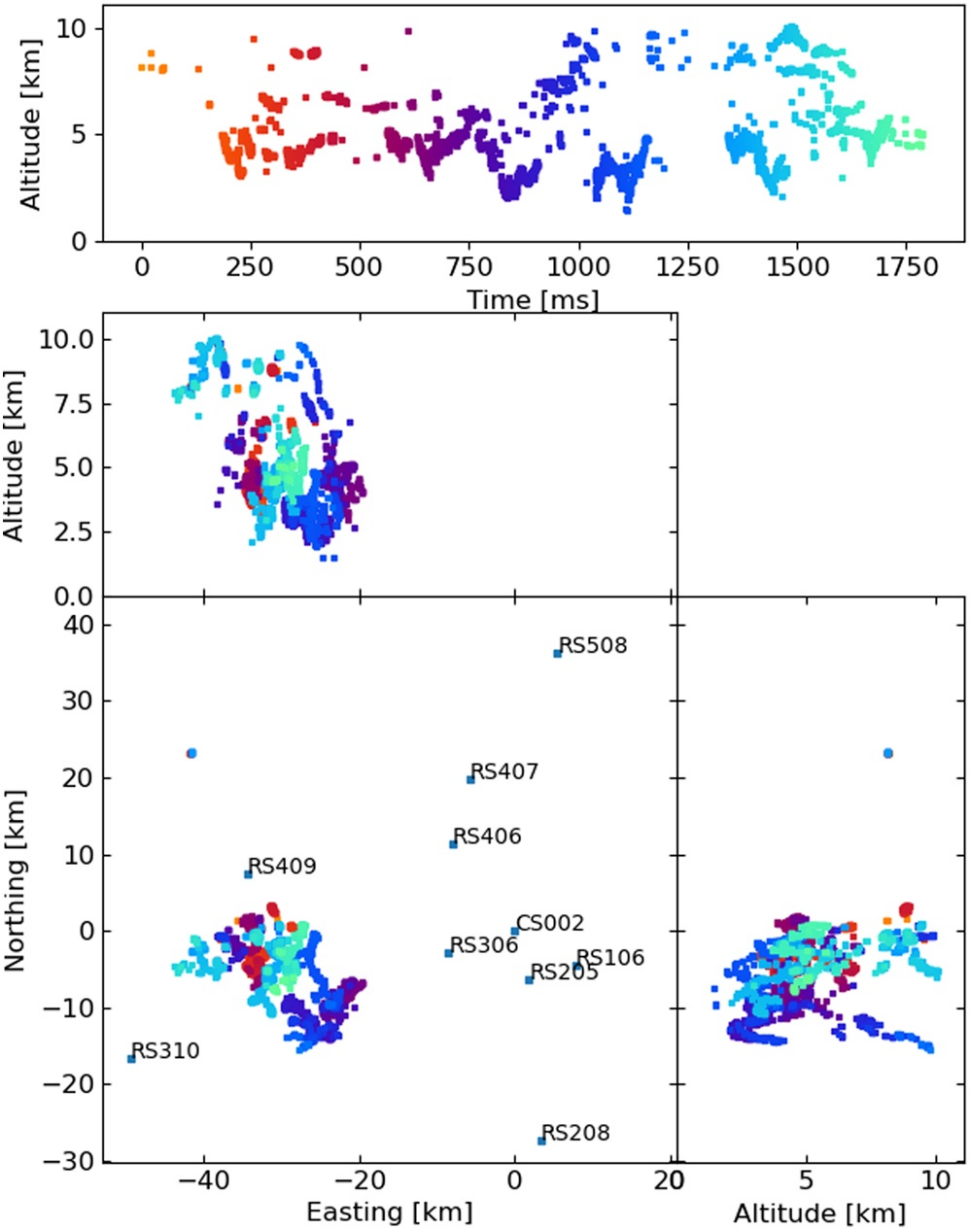
The 2019 lightning flash was mapped by the LOFAR LMA emulator using the traditional LMA windowing technique, along with the used LOFAR stations. Showing sources that have eight or more participating stations and a chi‐square value better than two (RMS < 21 ns).

The LOFAR LMA‐emulator uses data from two lightning flashes, one from 2018 and 2019, shown in Figures 1 and 2 respectively. For each flash, the stations were chosen to be as spread‐out as possible in order to best emulate the layout of an LMA. For each station, the data was band‐pass filtered between 60–66 MHz using a simple block filter, and the Hilbert envelope was found in order to emulate the log‐amplifier of the LMA (which outputs the logarithm of signal power from the LMA antenna). Then, for each 80 μs window aligned with the start of the second, the time of the highest peak was found, truncated to the nearest 40 ns, and saved to a file if the amplitude is greater than a noise threshold. Since LOFAR only saves five seconds of data, it is impossible to emulate the LMAs’ floating noise threshold, so instead noise thresholds were chosen visually. The times of the resulting pulses were then passed through the LMA processing algorithm. In Section 4, we test other windowing techniques to explore their effect. The LOFAR LMA‐emulator has a timing uncertainty of about 15 ns, which is dominated by the quantization of source arrival time when converting the LOFAR data into the LMA data format. After processing, the best‐located sources (located by all stations, with chi‐square values better than 1) have location errors in easting, northing, and altitude around 9, 21, 48, and 3, 2, and 20 m for the 2018 and 2019 flashes respectively. These location errors were calculated via the analytical covariance matrix.

## 4 Effect of Windowing on an LMA

In this section, we use the LOFAR LMA‐emulator to test the effect of different windowing techniques on LMA data and processing. First, we test the traditional binning technique with 80 μs wide windows that align with the start if the second. Then we test three new windowing techniques that we will refer to as “non‐aligned window”, “floating threshold”, and “natural threshold”. The details of these three windowing techniques are described below.

One challenge in this study was that the current LMA software implementation requires that there is only one recorded pulse per window (of either a 10  or 80 μs width), where the time of the window is fixed such that the windows align with the start and end of each second. Our new windowing techniques, however, must record multiple pulses per second‐aligned window, or else the result will be equivalent to the traditional windowing. To solve this we have designed our new windowing techniques to try to match the same average pulse rate of the traditional 80 μs window (that is, an average of 1 recorded pulse per 80 μs), but not have more than one recorded pulse per second‐aligned 10 μs window. We then processed all the data with the 10 μs mode of the LMA processing algorithm, including the traditional 80 μs window for consistency.

### 4.1 Non‐Aligned Window

The first new windowing technique is rather simple. A sample is recorded as a pulse if it has the highest amplitude within a ±40 μs region. Note that these windows can overlap, so two recorded pulses can be as close as 40 μs. We choose to test this method because the traditional windowing technique has a minimum time between pulses that varies randomly. This is because the times of the windows are fixed to be aligned with the start of the second as opposed to the time of the recorded pulse. Thus, since the recorded pulse can occur anywhere in a window, and the next recorded pulse can only occur as early as the start of the next window, then the minimum time between pulses is uniformly random from 0 up to the window width. This non‐aligned windowing technique, however, fixes the minimum time between pulses to be exactly 40 μs. The hope is that this improved consistency will allow the windowing technique to more reliably pick pulses that correspond with each other between the different stations. We expect, and show below, that a 40 μs minimum time, as opposed to a 80 μs, will result in about the same number of pulses as the traditional windowing. As the traditional windowing has an average minimum time of 40 μs (as it is uniformly distributed between 0 and 80 μs).

### 4.2 Floating Threshold

The goal of the second new windowing technique is to improve the ability of the LMA to handle bursts of pulses. That is, to allow the windowing technique to record pulses that are close together in time, but to have an amplitude threshold so that the average rate of pulses is similar to the traditional 80 μs windowing (an average of 1 recorded pulse per 80 μs).

This is done by implementing a floating threshold similar to the floating noise threshold already present in LMAs, but shortened to work on a smaller timescale. To do this, we track the highest sample in 10 μs bins, similar to the traditional 10 μs windowing. However, this sample is only recorded to file if its amplitude is larger than a threshold that is adjusted every 400 μs. If there are more than five recorded pulses in the previous 400 μs then the threshold is increased by 10%, if there are less than five then the threshold is decreased by 10%. Note that each 400 μs period is consecutive and not overlapping, because if the periods overlap then this technique will become unstable and the threshold will oscillate up and down even when the pulse amplitude distribution in the data is constant. A noise threshold is still implemented, and any pulse that has an amplitude below the noise threshold is discarded.

### 4.3 Natural Threshold

Our final windowing technique has a similar goal to the floating threshold, in that we want to be able to record pulses that occur close together in time while maintaining an average rate of 80 μs, which we accomplish with a dynamic amplitude threshold. The difference between this technique and the floating threshold, is that this technique has the additional goal that we do not want to explicitly track this amplitude threshold. Instead, we want a simple technique that only considers data centered on each pulse completely independently, not relying on memory of which pulses were previously recorded to file.

We have accomplished this by first finding the highest sample in 10 μs bins, again like the traditional LMA windowing, but we do not record this pulse. Instead, we save it into a circular buffer of 80 10 μs bins (a total width of 800 μs). The sample in the 40th bin (that is the sample in the middle of our buffer) is recorded to file if no more than 9 other bins contain stronger pulses. i.e., a pulse is saved to file if it is one of the top 10 strongest pulses in 800 μs (centered on that pulse) and it is above the noise threshold. This results in an average of 10 pulses recorded per 800 μs, and the decision of whether or not a pulse is recorded is entirely independent of whether or not any other pulse is saved.

This natural threshold windowing technique has one potential drawback over the traditional 80 μs windowing technique. This is due to the possibility that a lightning process could produce a very strong VHF burst that lasted around 100 μs long. If this occurred, then the natural threshold window would saturate on just that VHF burst and would not record any other VHF emissions for ±400 μs centered around that burst. The traditional 80 μs windowing technique does not present this problem.

### 4.4 Results

Figures 3, 4, 5, 6 show results for the four windowing techniques for the 2018 flash, zoomed in to a well‐imaged negative leader. Sources with eight or more participating stations are shown. Figures 7, 8, 9, 10 are similar for the 2019 flash.

**Figure 3:**
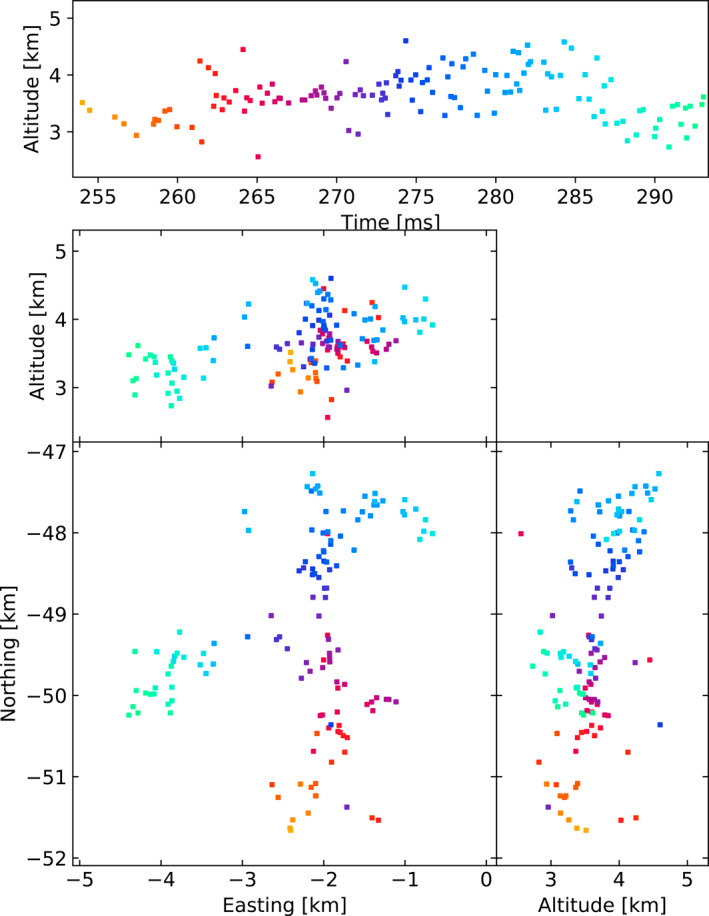
80 μs traditional windowing with the LMA‐emulator, centered on a negative leader in the 2018 flash. Showing 134 sources that have eight or more participating stations.

**Figure 4:**
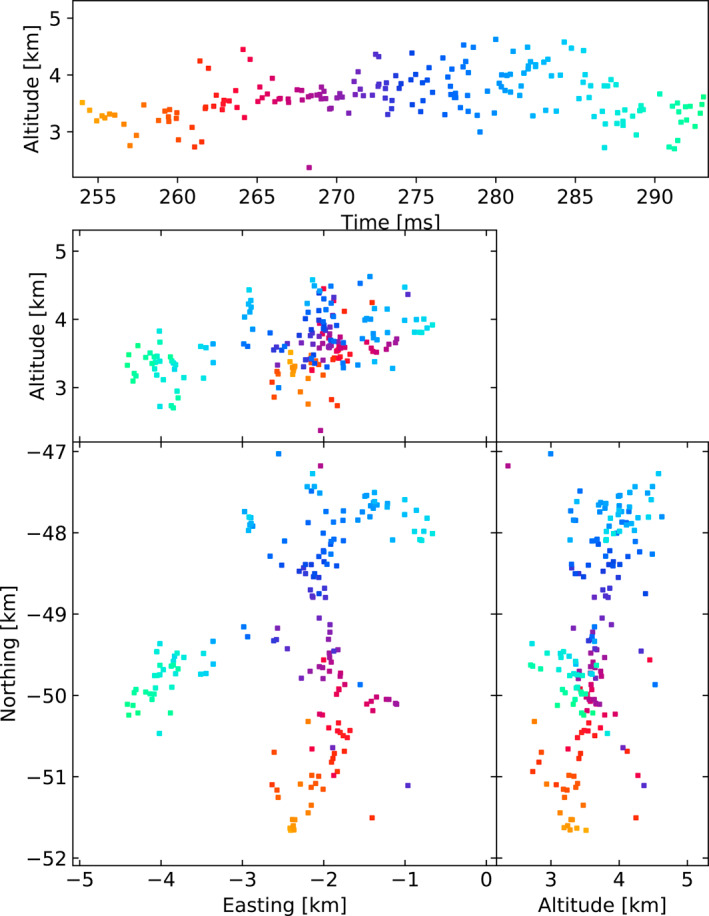
Natural threshold windowing with the LMA‐emulator, centered on a negative leader in the 2018 flash. Showing 181 sources that have eight or more participating stations.

**Figure 5:**
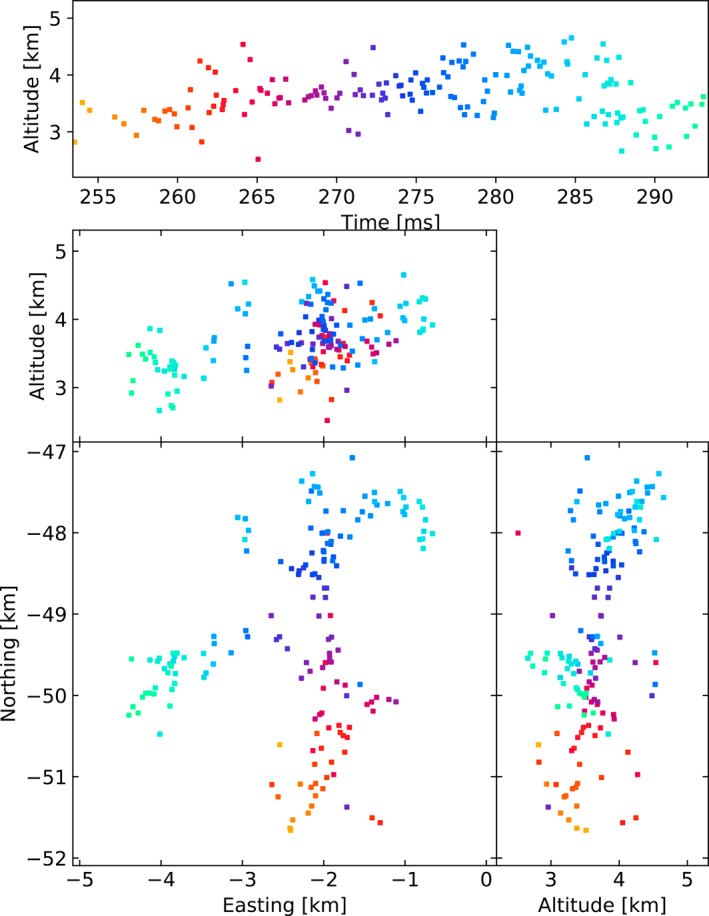
Non‐aligned windowing with the LMA‐emulator, centered on a negative leader in the 2018 flash. Showing 164 sources that have eight or more participating stations.

**Figure 6:**
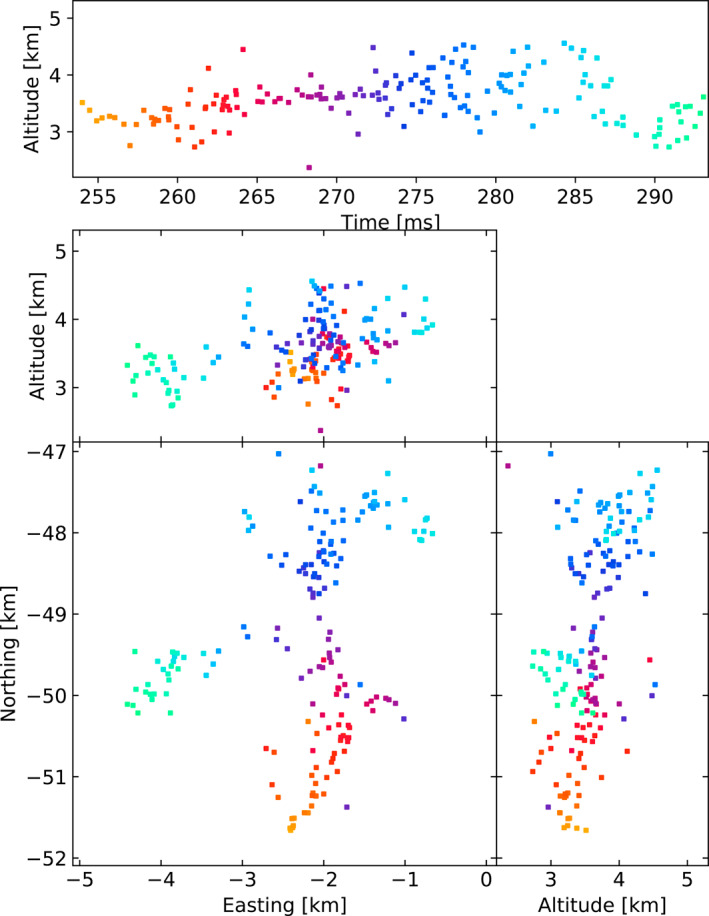
Floating threshold windowing with the LMA‐emulator, centered on a negative leader in the 2018 flash. Showing 173 sources that have eight or more participating stations.

**Figure 7:**
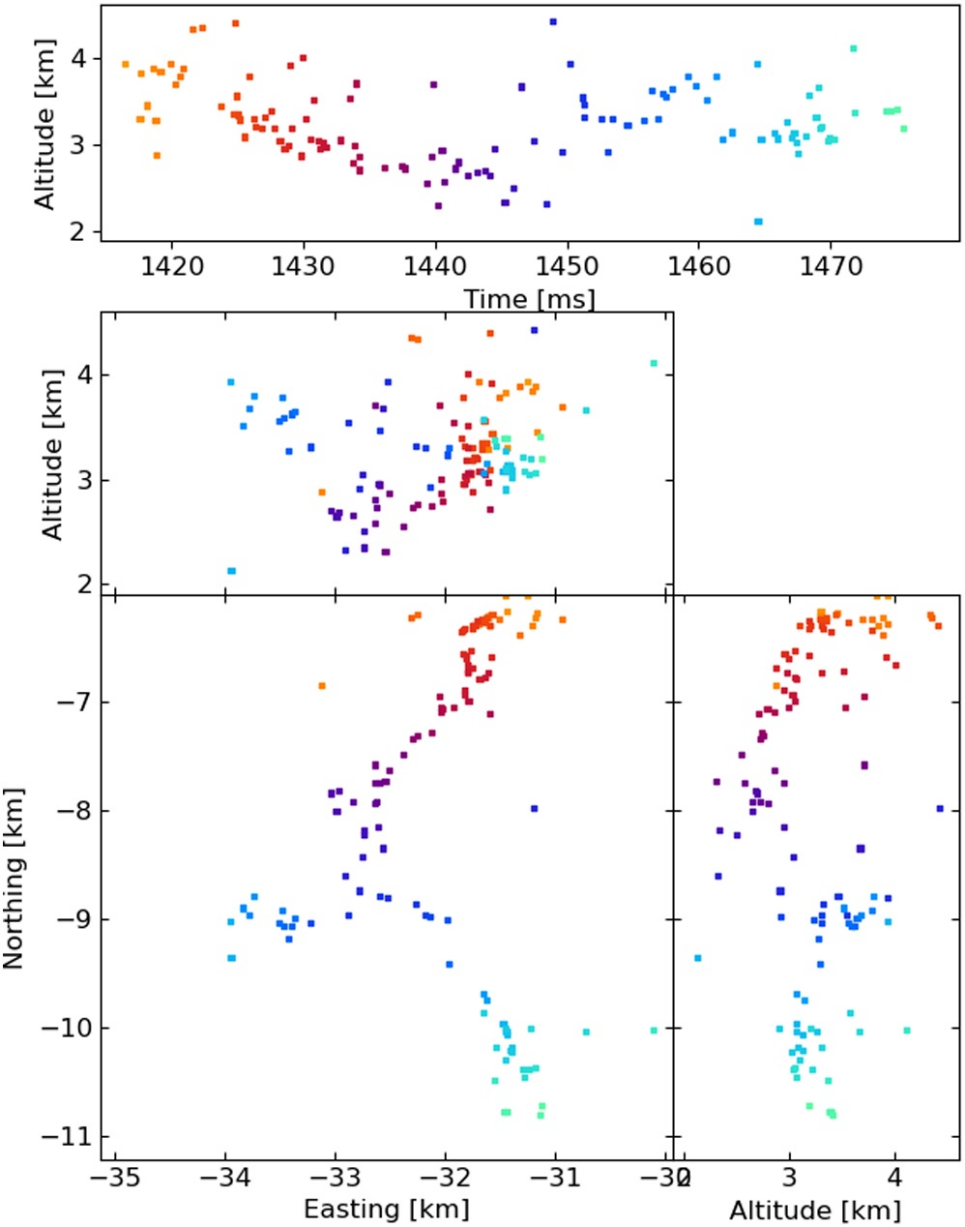
80 μs traditional windowing with the LMA‐emulator, centered on a negative leader in the 2019 flash. Showing 117 sources that have eight or more participating stations.

**Figure 8:**
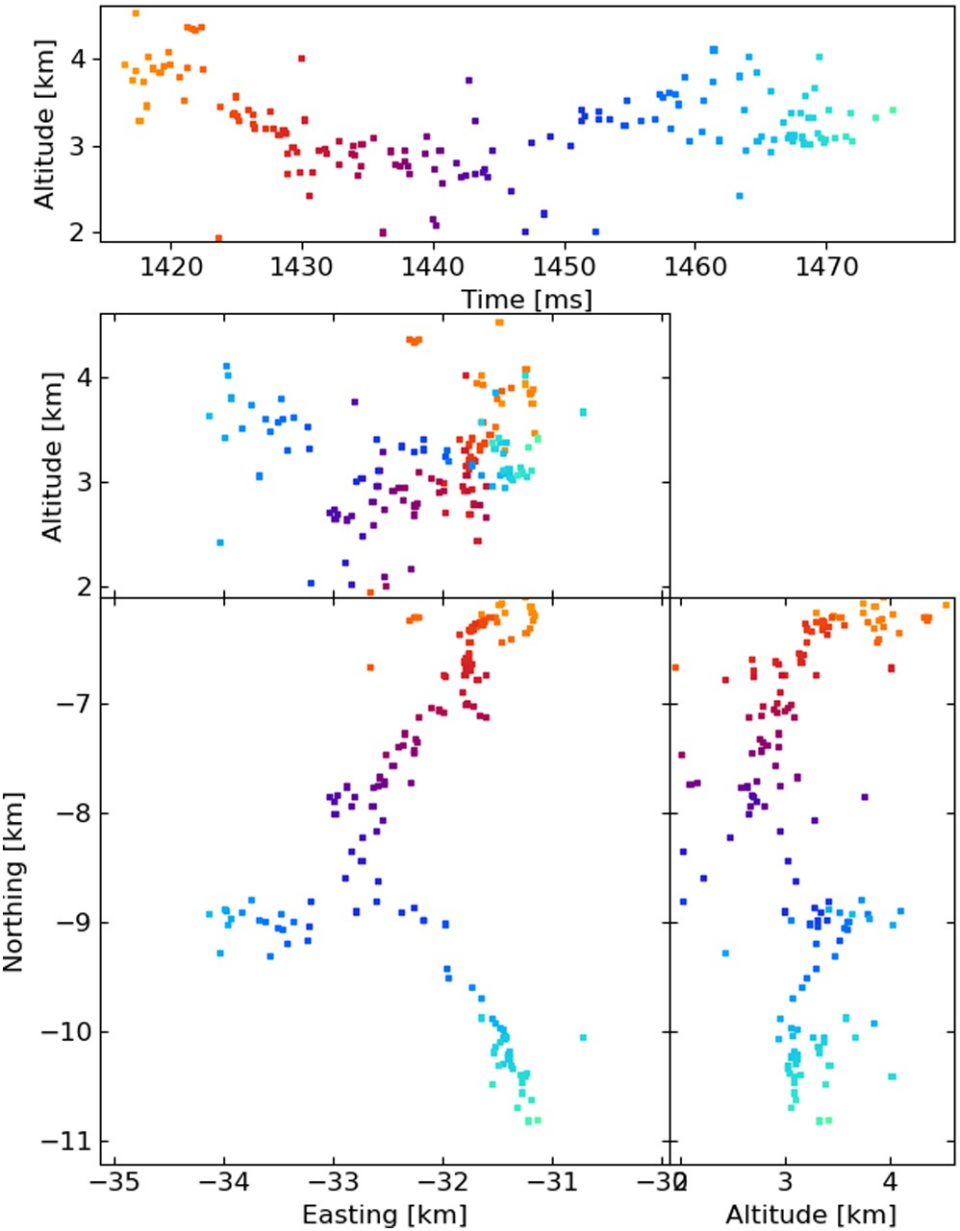
Natural threshold windowing with the LMA‐emulator, centered on a negative leader in the 2019 flash. Showing 152 sources that have eight or more participating stations.

**Figure 9:**
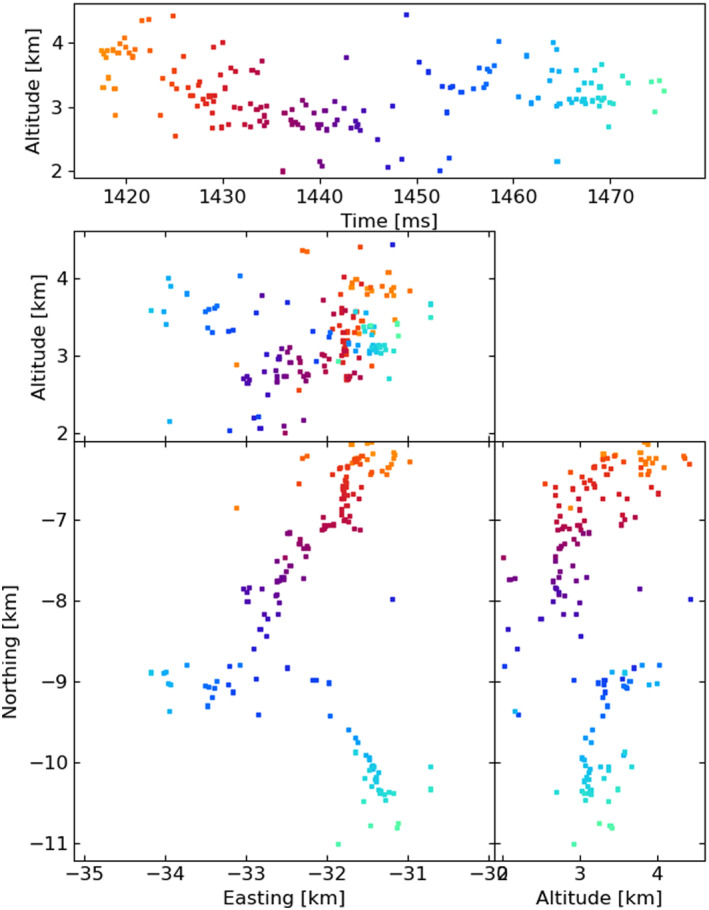
Non‐aligned windowing with the LMA‐emulator, centered on a negative leader in the 2019 flash. Showing 154 sources that have eight or more participating stations.

**Figure 10:**
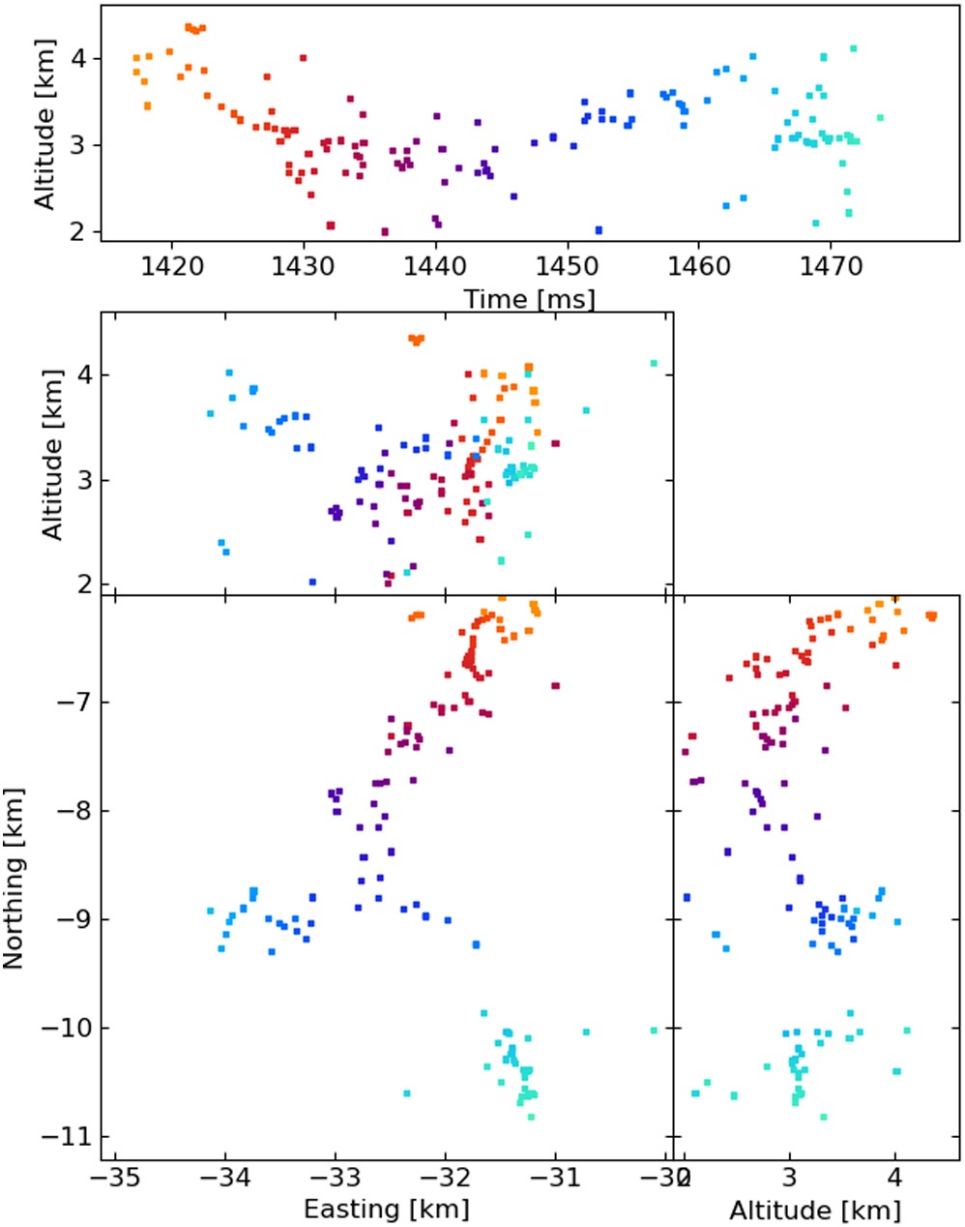
Floating threshold windowing with the LMA‐emulator, centered on a negative leader in the 2019 flash. Showing 130 sources that have eight or more participating stations.

Some statistical results for each windowing technique are shown in Tables 1 and 2 for the 2018 and 2019 flashes respectively. The row “average pulses per station” gives the number of pulses recorded with the relevant method averaged over all 10 LOFAR stations, over the absolute theoretical maximum number of pulses that could have been recorded. Row “relative pulse number difference” gives the difference between the minimum and the maximum number of pulses recorded for each station, divided by the average recorded pulses per station, in order to give a measure of the deviation of recorded pulses between stations. As one would expect, stations farther from the flash recorded fewer pulses. Next, there are four sets of two rows. These are two statistics for four different cuts of sources. The four sets of cuts are: 1) no cuts (all sources output by the LMA processing algorithm), 2) sources that have eight or more participating stations, 3) sources that pass cut 2 and have a chi‐square value less than 2 (RMS < 21 ns), finally 4) sources that pass cut 3 and are in the vicinity of the imaged flash. For each of the four cuts, we list the number of sources, and the ratio between the number of sources and the number of recorded pulses.

**Table 1:** Results of Different Windowing Techniques for the 2018 Flash

Statistic	80 μs windows	Non‐aligned windows	Floating threshold	Natural threshold
Average recorded pulses/maximum pulses Relative pulse number difference	6,450/8,800 0.50	6,340/8,800 0.48	9,032/70,500 0.62	6,944/70,500 0.39
Number sources (no cut) Source/pulse ratio	4,120 0.64	4,592 0.72	7,552 0.84	5,222 0.75
Number sources (Cut 1) Source/pulse ratio	1,905 0.29	2,257 0.35	2,796 0.31	2,729 0.39
Number sources (Cut 2) Source/pulse ratio	1,489 0.23	1,749 0.27	2,078 0.23	2,092 0.30
Number sources (Cut 3) Source/pulse ratio	1,466 0.23	1,722 0.27	2,044 0.23	2,059 0.30

**Table 2:** Results of Different Windowing Techniques for the 2019 Flash

Statistic	80 μs windows	Non‐aligned windows	Floating threshold	Natural threshold
Average recorded pulses/maximum pulses	13,180/22,500	12,730/22,500	21,860/180,200	14,260/180,200
Relative pulse number difference	0.38	0.36	0.62	0.32
Number sources (no cut)	8,123	9,011	20,700	11,030
Source/pulse ratio	0.62	0.70	0.95	0.77
Number sources (Cut 1)	3,226	3,997	3,561	4,006
Source/pulse ratio	0.24	0.31	0.16	0.28
Number sources (Cut 2)	1,687	2,053	1,890	2,121
Source/pulse ratio	0.13	0.16	0.09	0.15
Number sources (Cut 3)	1,675	2,041	1,879	2,109
Source/pulse ratio	0.13	0.16	0.09	0.15

As discussed in Section 2, the timing uncertainty of an LMA data set can be found by matching the calculated reduced chi‐square distribution with the expected reduced chi‐square distribution. Doing so we found that the timing uncertainty for all four windowing techniques was 14 ns. In other words, the windowing technique does not seem to affect the timing uncertainty for our LMA‐emulator. The 14 ns uncertainty is essentially the quantization uncertainty of 25 MHz digitization discussed in Sections in 2 and 3.

From these results, we can see that the four windowing techniques produce similar results. A comparison between the images shown in Figures 3, 4, 5, 6 for the 2018 flash and Figures 7, 8, 9, 10 for the 2019 flash show that each of the windowing techniques shows the same general features on the 100 m scale.

Table 1 shows that, for the 2018 flash, the non‐aligned windows give a nearly identical result to the traditional LMA windowing. The natural threshold, on the other hand, has a slightly improved (≈10%) ratio between located events over received pulses. The floating threshold saved significantly more pulses than the other windowing techniques, despite being designed to have the same rate. The reason for recording more pulses than intended is because the amplitude distribution of pulses in a lightning flash can change very quickly, which makes it very difficult to design a floating threshold that behaves predictably. Table 2 shows very similar results for the 2019 flash. It also shows that every windowing technique had significantly lower ratios between located sources and detected pulses during the 2019 flash as compared to the 2018 flash. It is presently unknown why different flashes result in different processing efficiencies.

Table 1 shows that, for the 2018 flash, the non‐aligned windows record slightly less pulses then the traditional LMA windowing, but results in slightly more located events. The natural threshold saved about 8% more pulses, but was able to located about 40% more events. The floating threshold saved significantly more pulses than the other windowing techniques, despite being designed to have the same rate. The reason for recording more pulses than intended is because the amplitude distribution of pulses in a lightning flash can change very quickly, which makes it very difficult to design a floating threshold that behaves predictably. Table 2 shows very similar results for the 2019 flash. It also shows that every windowing technique had significantly lower ratios between located sources and detected pulses during the 2019 flash as compared to the 2018 flash. It is presently unknown why different flashes result in different processing efficiencies.

**Figure 11:**
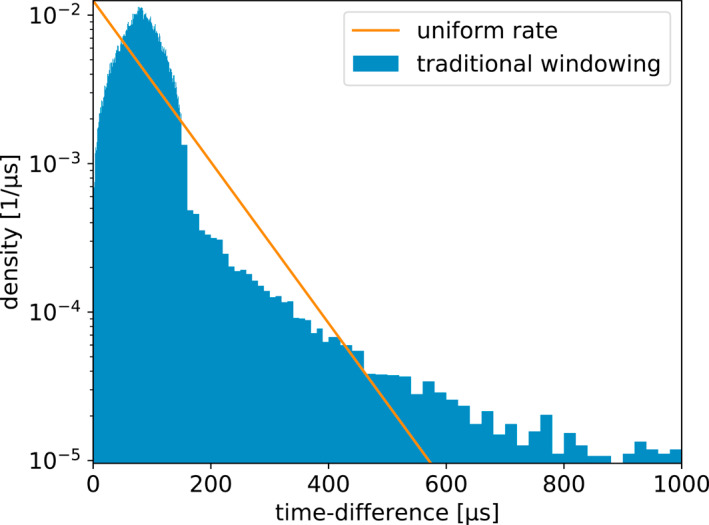
Distribution of time between saved pulses for the traditional 80* *μs windowing technique. The line shows the expected distribution if the pulses were saved at a random rate of one per 80 μs.

**Figure 12:**
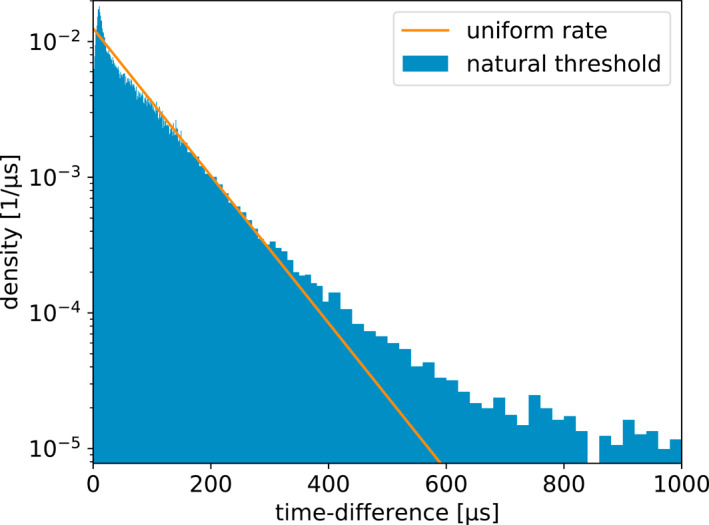
Distribution of time between saved pulses for the natural threshold windowing technique. The line shows the expected distribution if the pulses were saved at a random rate of one per 80 μs.

**Figure 13:**
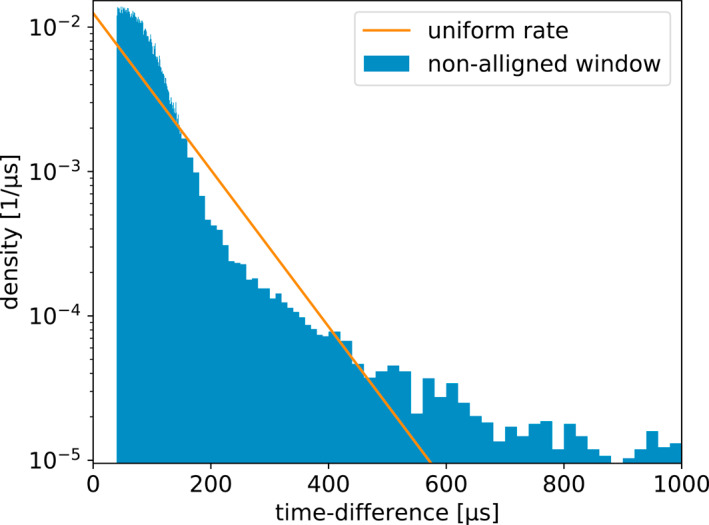
Distribution of time between saved pulses for the non‐aligned windowing technique. The line shows the expected distribution if the pulses were saved at a random rate of one per 80 μs.

**Figure 14:**
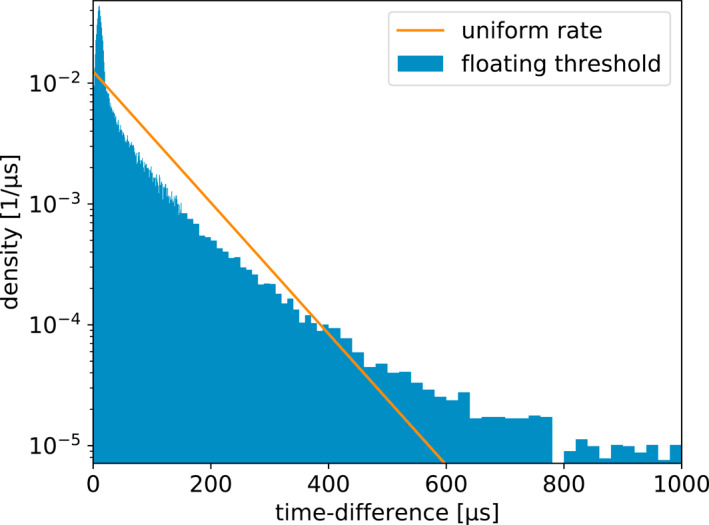
Distribution of time between saved pulses for the floating threshold windowing technique. The line shows the expected distribution if the pulses were saved at a random rate of one per 80 μs.

It is interesting to compare the distributions of time between recorded pulses for each of the four windowing techniques. This is shown in Figures 11, 12, 13, 14 for the 2019 flash. These figures also show a straight line, which is the expected distribution if pulses were recorded with a random independent rate of one per 80 μs. The same distributions for the 2018 flash look extremely similar. As one would expect, the distribution of times between recorded pulses for traditional binning, shown in Figure 11, has a symmetrical peak around 80 μs with a tail extending to longer time scales. The natural threshold, shown in Figure 12 has an extremely good match to an independent random rate, as designed. However, it has a spike at time‐differences of 10 μs, which could be due to the fact that this technique still uses 10 μs binning at its core to operate. Non‐aligned binning, shown in Figure 13, is similar to the traditional windowing, except that there are no pulses closer than 40 μs and the distribution has a smoother transition between the central peak and tail (starting at about 160 μs). Finally, the distribution produced by the floating threshold is shown in Figure 14, which has a very strong peak at around 10 μs, followed by a fairly regular rate that is lower than one per 80 μs.

## 5 Timing Calibration

In this section, we apply the calibration technique that we developed for LOFAR, to the LMA data. This calibration technique is capable of finding any relative timing offset between LMA stations, including the GPS timing offsets.

### 5.1 The Algorithm

The fundamental idea behind our calibration algorithm is that the time between pulses of different sources on each antenna, even if the absolute time is unknown, is enough to constrain the source location. This information is used simply by fitting the arrival times of pulses from multiple sources, where the fitting parameters are the location and time of each source and the relative timing offset of all but one station. This is expressed through Equations 3 and 4, where Equation 3 is the modeled arrival time given the source locations and relative station delays, and Equation 4 is the reduced chi‐square.

(3) $$M_{i,j}=\frac{\sqrt{{\left(x_{i}-x_{j}\right)}^{2}+{\left(y_{i}-y_{j}\right)}^{2}+{\left(z_{i}-z_{j}\right)}^{2}}}{C}+t_{i}+\Delta t_{j}$$

where *M*_*i*,*j*_ is the calculated arrival time for the *i*th source on the *j*th antenna. *x*_*i*_, *y*_*i*_, *z*_*i*_, and *t*_*i*_ is the location and time of the *i*th source. *x*_*j*_, *y*_*j*_, *z*_*j*_, and Δ*t*_*j*_ is the location and time delay of *j*th antenna. *C* is the speed of light. The fitted parameters are *x*_*i*_, *y*_*i*_, *z*_*i*_, *t*_*i*_, and Δ*t*_*j*_, which are the locations and times of the sources, and the time delays of the antennas. Note that the time delay for one station, the reference station, is held to 0. Given this point source model, the reduced chi‐square can be calculated,

(4) $$\chi^{2}\left(x_{i},y_{i},z_{i},\;t_{i},\;\Delta t_{j}\right)=\frac{1}{N_{m}-N_{a}-4N_{s}}\sum_{i,j}\frac{{\left(M_{i,j}-t_{i,j}\right)}^{2}}{\sigma_{\epsilon }^{2}}$$

where *χ* ^2^ is the reduced the chi‐square. *N*_*m*_ is the number of measurements, that is, the sum of number of active antennas used in locating each source. *N*_*s*_ is the number of sources fitted. *N*_*a*_ is the number of antennas. *t*_*i*,*j*_ is the measured arrival time of source *i* on antenna *j*. Note this sum skips *i*, *j* combinations when the *i*th source is not detected on the *j*th antenna. The decision of which pulse to use in locating an event, and whether or not to exclude a station entirely is made by the LMA processing algorithm. Finally, *σ*_*ϵ*_ is the estimated timing uncertainty.

The difference between this technique and normal LMA source locating is that multiple sources are fit simultaneously, and the relative time calibrations between stations are fitted parameters.

The difficulty in any time‐of‐arrival algorithm is deciding which measured pulse times to associate with which source. For the LMA this problem already has a solution in the LMA data processing program. This program, fortunately, also saves the times of the pulses associated with each source accounting for known delays. Thus, our algorithm is designed to be applied to processed LMA data. The resulting delays can then be fed back into the LMA processing code in order to produce a better image. However, since the LMAs’ systematic timing delays change at the beginning of every second, via the GPS updating the station clock, our algorithm has to be applied separately to each second of LMA data.

Each calibration run uses between 20 and 50 LMA sources and their associated pulses to find the locations, times, and antenna delays that minimize the *χ* ^2^ value, via a Levenberg‐Marquardt minimizer. In order to estimate the uncertainty of the extracted relative timing delays, this procedure is run multiple times on different sets of sources. We sort the LMA sources so that, for the number of runs (*N*_*r*_) there are at least *N*_*p*_ sources on each antenna. The extracted delays are than the average of the runs, and the estimated uncertainties are the standard deviation of the runs divided by the square root of number of runs. The LMA sources used in the calibration were chosen by picking LMA sources at random that have RMS fit values better than the timing uncertainty of the LMA network, and a minimal number of participating stations. We purposefully do not pick LMA sources with the absolute best fit values, as they tend to have random uncertainty that cancel‐out the systematic uncertainties that we wish to extract. The full procedure is shown in Algorithm 1.

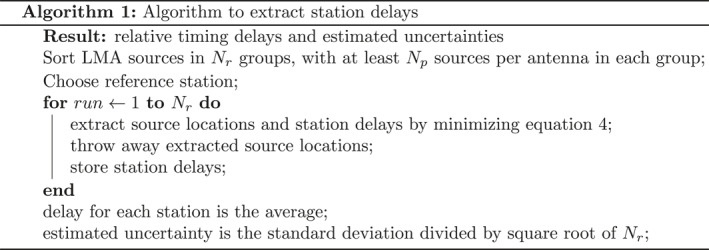

### 5.2 Calibration Test With the LOFAR LMA‐Emulator

The LOFAR LMA‐Emulator presents a perfect platform for which to test our calibration algorithm, since the LOFAR data has already been calibrated such that any systematic uncertainty (∼1 ns) is much smaller than the random uncertainty inherent in the LMA emulator (∼12 ns). In order to perform this test, we ran the LOFAR LMA‐emulator to obtain a set of LMA sources based on lightning data recorded by LOFAR. We then injected a systematic delay to the pulses recorded by each station. These injected systematic delays were drawn independently from a normal distribution with 10 ns standard deviation. We then ran our calibration technique, Algorithm 5.1, and attempted to re‐extract the injected delays with expected uncertainties. Note that this test injects the systematic timing uncertainty after the LMA location algorithm, where, in reality, the systematic timing uncertainties are injected before the LMA location algorithm. The implication is, in a more realistic situation the offsets we wish to find could cause the LMA location algorithm to associate the wrong pulse with an event. Thus, since we rely on the LMA location algorithm to pick which pulses to associate with each event, real data could result in a somewhat lower quality calibration than indicated by this test.

The injected delays, extracted delays, estimated uncertainties and actual uncertainties are shown in Table 3. Note that, despite using 10 stations, only nine are shown in Table 3 since both the injected and estimated uncertainty were held to zero on the reference station, which was CS002.

**Table 3:** Results of Applying the Calibration Algorithm to the LMA‐Emulator, Where CS002 was the Reference Station

Station	Injected delay [ns]	Extracted delay [ns]	Estimated uncertainty [ns]	Actual error [ns]
CS002	0.0	0.0	0.0	0.0
RS205	−1.6	0.0	1.0	−1.6
RS306	−7.1	−7.0	0.8	−0.1
RS406	−11.9	−15.2	0.9	3.3
RS307	17.2	18.5	1.2	−1.3
RS407	7.7	7.9	1.5	−0.2
RS409	−10.6	−11.4	0.8	0.8
RS208	−20.0	−20.4	0.9	0.4
RS508	6.6	7.6	0.9	−1.0
RS310	−8.9	−8.3	0.6	−0.6

Table 3 shows that the extracted time delays are very similar to the injected time delay, and that the estimated uncertainties in general reflect the actual uncertainties. In this particular run there is one station, RS406, where the difference between the extracted delay and injected delay is 3–4 times that of the estimated uncertainty. This, however, is not surprising, as the estimated uncertainty is probably only accurate to a factor of 2.

### 5.3 Application of Calibration to COLMA

We have applied our new calibration algorithm to 600 s of data from the COLMA. We found that the timing uncertainty improved from 32 to 19 ns. This represents a significant improvement, however, it is known that the random uncertainty of the LMA should be around 12 ns. Indeed, the LOFAR LMA‐emulator, which attempts to emulate the dominant random uncertainty sources of the LMA, has a timing uncertainty of about 15 ns. Thus, we would expect the post‐calibration timing uncertainty to be better than 19 ns, and it is not clear why this is not the case. One possibility is that the calibration algorithm used poorly reconstructed LMA sources, where pulses from different real VHF sources were associated with each other, which could have biased the result.

Each of these 600 s were processed independently, and since the timing of the LMA stations is updated every second the resulting timing calibrations will be different for every second according to the timing error of the LMA. In order to explore this second‐to‐second calibration variation, Table 4 reports the average extracted delay which should be zero (within statistical fluctuations) if each station has no unaccounted systematic timing delay. The second column of Table 4 shows the standard deviation of the extracted delay, which is due to (and should be similar to) the LMA timing error (32 ns). These standard deviations do not reflect the accuracy of the calibrations. The final column gives the estimated uncertainty of the average (calculated through standard deviation divided by the square root of the number of samples). Since every processed second can use a different reference station, we only used extracted delays that had the same reference station (Rodenburg) in order to calculate the statistics in Table 4. Out of the 600 s of processed LMA data, our algorithm used the Rodenburg station for 58 of the processed seconds. Therefore 58 samples were used to derive the statistics shown in Table 4.

**Table 4:** Average, Standard Deviation, and Standard Error of the Mean From Applying the Calibration Procedure to COLMA Data, Where Rodenburg was the Reference Station

COLMA station	Average delay [ns]	Delay standard deviation [ns]	Uncertainty of the average [ns]
Rodenburg	0.0	0.0	0.0
Briggsdale	−21.2	32.0	4.2
LoneTree	−6.5	55.3	7.3
GreeleyArpt	−12.1	18.0	2.4
Raymer	33.3	57.1	7.5
FtCollinsArpt	−1.8	25.0	3.3
Herford	−9.5	62.3	8.1
Homestead	−18.3	61.3	8.0
Purcell	4.7	37.4	4.8
CPER	−22.0	46.0	6.0
WeldCHS	3.0	17.1	2.2
ButteEdge	5.3	54.0	7.1
Boyer	−0.1	43.8	5.8
FMA	−15.8	35.2	4.6
WigginsHS	8.1	31.5	4.2

The standard deviations are about 32 ns, which is consistent with the known timing error of this set of COLMA data. A few stations have large average delays (greater than three times the uncertainty). This implies that the COLMA LMA has significant un‐accounted‐for systematic relative delays other than GPS‐related timing offsets. The source of these systematic relative delays is not clear, as all COLMA stations use the same cable lengths in order to minimize this exact problem. More work would be needed to explore if this is indeed the case, and what the cause of these systematic offsets could be.

**Figure 15:**
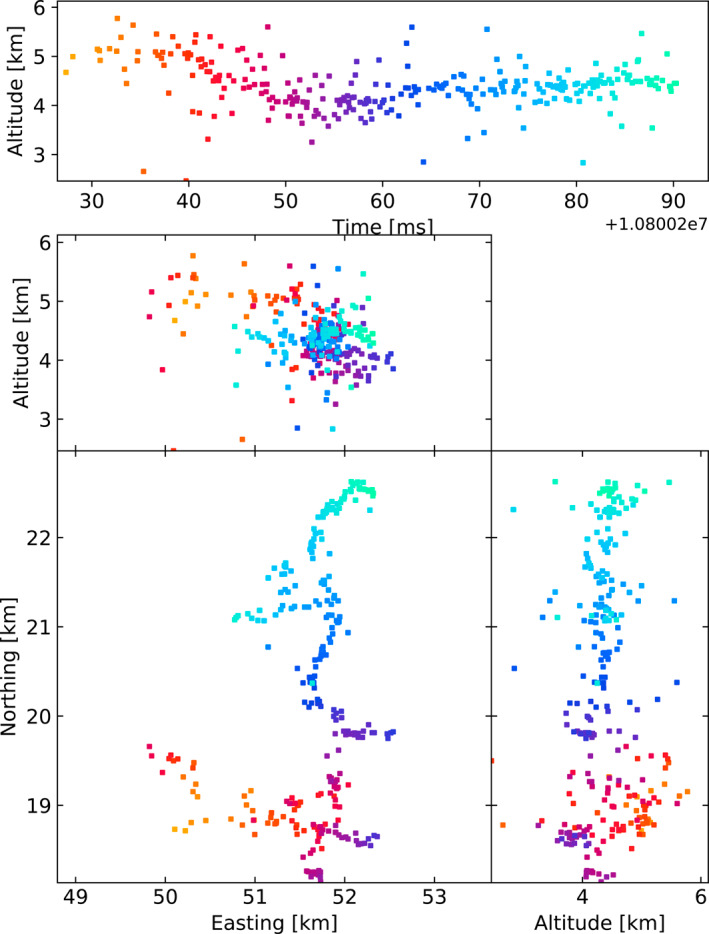
Negative leader imaged by COLMA before calibration. 280 sources with eight or more participating stations are shown.

**Figure 16:**
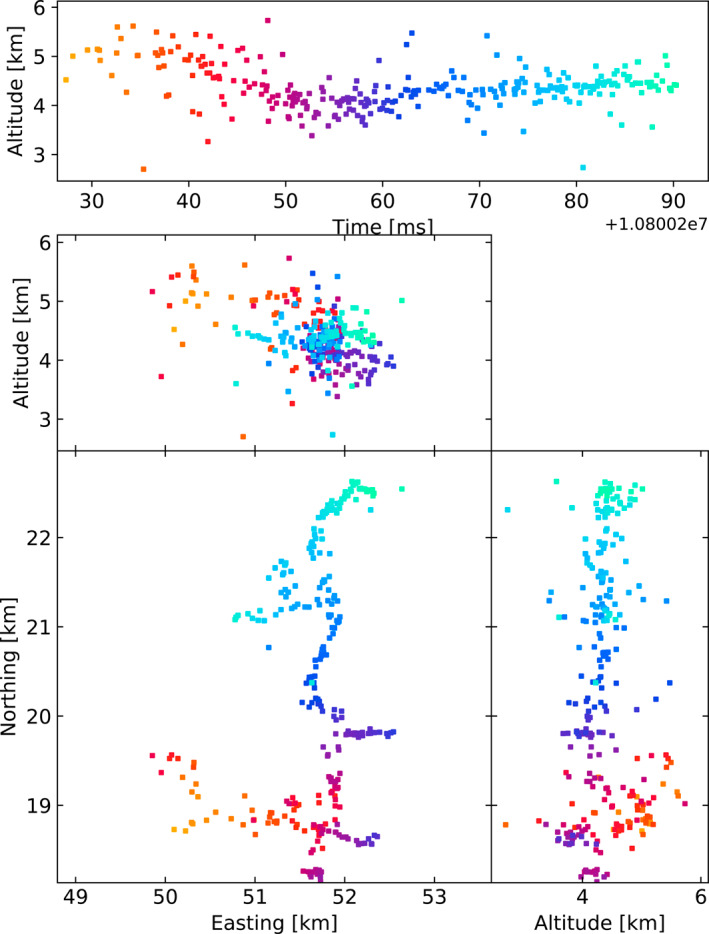
Negative leader imaged by COLMA after calibration. 286 sources with eight or more participating stations are shown.

Figures 15 and 16 show a negative leader imaged by COLMA before and after calibration respectively. These figures show 280 and 286 sources that have eight or more participating stations respectively. These figures show that, despite the significant improvement in timing, there is little improvement in image quality as the two images are extremely similar.

## 6 Conclusion

In this work, we developed a system to emulate the operation of an LMA with LOFAR. This LMA‐emulator allowed us to test the effect of different windowing techniques. We tested three new windowing techniques and compared them to the traditional LMA windowing. We found that these more sophisticated windowing techniques result in images that are, by eye, not obviously improved over the older simpler technique. This shows that lightning physics extracted using LMAs is not sensitive to the windowing technique used.

In addition, we have developed a new calibration technique, based on our experience with calibrating LOFAR, that can extract relative systematic timing delays between LMA stations on a second‐by‐second basis. Using this calibration technique we were able to reduce the timing uncertainty of 600 s of data collected from COLMA from 32 to 19 ns, when COLMAs’ typical timing uncertainty is about 25 ns.

Despite the modest improvements of these techniques to the LMA data, we believe that this work has three important implications. First, by demonstrating the unique flexibility of the LOFAR instrument. At the moment LOFAR is operated in a triggered mode, to produce high‐quality images of a few flashes (e.g., (Hare et al., 2019; Scholten et al., 2021)). However, this work suggests the possibility of operating LOFAR in a continuous LMA‐like mode to capture every lightning flash in a storm with lower quality, which will be investigated in future work. Second, this work testifies to the robustness of the LMA system and processing algorithm, that even significant changes to the processing technique do not result in noticeable differences in the reconstructed lightning. Finally, and perhaps most critically, this work establishes two new post‐processing techniques that can be applied to almost any lightning‐mapping system, not just LMAs. This is especially important for multi‐station lightning interferometers, which have proven to be very difficult to calibrate without this technique (Hare et al., 2018; Jensen et al., 2021).

## Data Availability

The data used in this work is available at (Hare et al., 2020).
